# High frequency hearing thresholds and product distortion otoacoustic emissions in cystic fibrosis patients^☆^^☆☆^

**DOI:** 10.1016/j.bjorl.2015.08.011

**Published:** 2015-09-08

**Authors:** Lucia Bencke Geyer, Sergio Saldanha Menna Barreto, Liese Loureiro Weigert, Adriane Ribeiro Teixeira

**Affiliations:** a Hospital de Clínicas de Porto Alegre, Porto Alegre, RS, Brazil; b Child and Adolescent Health, Universidade Federal do Rio Grande do Sul (UFRGS), Porto Alegre, RS, Brazil; c Toronto University, Toronto, Canada; d Biomedical Gerontology, Pontifícia Universidade Católica do Rio Grande do Sul (PUC-RS), Porto Alegre, RS, Brazil; e Department of Human Health and Communication, Universidade Federal do Rio Grande do Sul (UFRGS), Porto Alegre, RS, Brazil

**Keywords:** Cystic fibrosis, Aminoglycosides, Audiometry, Fibrose cística, Aminoglicosídeos, Audiometria

## Abstract

**Introduction:**

The treatment of patients with cystic fibrosis involves the use of ototoxic drugs, mainly aminoglycoside antibiotics. Due to the use of these drugs, fibrocystic patients are at risk of developing hearing loss.

**Objective:**

To evaluate the hearing of patients with cystic fibrosis by High Frequency Audiometry and Distortion Product Otoacoustic Emissions.

**Methods:**

Cross-sectional study. The study group consisted of 39 patients (7–20 years of age) with cystic fibrosis and a control group of 36 individuals in the same age group without otologic complaints, with normal audiometric thresholds and type A tympanometric curves. High Frequency Audiometry and Distortion Product Otoacoustic Emissions tests were conducted.

**Results:**

The study group had significantly higher thresholds at 250, 1000, 8000, 9000, 10,000, 12,500, and 16,000 Hz (*p* = 0.004) as well as higher prevalence of otoacoustic emission alterations at 1000 and 6000 Hz (*p* = 0.001), with significantly lower amplitudes at 1000, 1400, and 6000 Hz. There was a significant association between alterations in hearing thresholds in High Frequency Audiometry with the number of courses of aminoglycosides administered (*p* = 0.005). Eighty-three percent of patients who completed more than ten courses of aminoglycosides had hearing loss in High Frequency Audiometry.

**Conclusion:**

A significant number of patients with cystic fibrosis who received repeated courses of aminoglycosides showed alterations in High Frequency Audiometry and Distortion Product Otoacoustic Emissions. The implementation of ten or more aminoglycoside cycles was associated with alterations in High Frequency Audiometry.

## Resumo

**Introdução:**

O tratamento dos pacientes com fibrose cística envolve o uso de medicamentos ototóxicos, principalmente de antibióticos aminoglicosídeos. Devido ao uso destes medicamentos, os pacientes fibrocísticos apresentam risco de desenvolver perda auditiva.

**Objetivo:**

Avaliar a audição dos pacientes com fibrose cística pela Audiometria de Altas Frequências (AAF) e Emissões Otoacústicas por Produto de Distorção (EOAPD).

**Método:**

Estudo transversal. O grupo de estudo foi constituído por 39 pacientes com idades entre 7 e 20 anos com fibrose cística e o grupo controle por 36 indivíduos da mesma faixa etária, sem queixas otológicas, com limiares audiométricos normais e curvas timpanométricas tipo A. Foram realizados os exames de audiometria de altas frequências e emissões otoacústicas por produto de distorção.

**Resultados:**

O grupo de estudo apresentou limiares significativamente mais elevados em 250; 1000; 8.000; 9.000; 10.000; 12.500 e 16.000 Hz; (*p* = 0,004) e maior prevalência de alterações nas emissões otoacústicas em 1.000 e 6.000 Hz (*p* = 0,001); com amplitudes significativamente mais baixas em 1.000; 1.400 e 6.000 Hz. Houve associação significativa entre as alterações dos limiares auditivos na AAF com o número de ciclos de aminoglicosídeos realizados (*p* = 0,005). Oitenta e três por cento dos pacientes que foram submetidos a mais de 3 ciclos de aminoglicosídeos apresentaram perda auditiva na AAF.

**Conclusão:**

Um número significativo de pacientes com fibrose cística que receberam repetidos ciclos de aminoglicosídeos apresentou alterações na AAF e EOAPD. A realização de 10 ou mais ciclos de aminoglicosídeos esteve associada às alterações na AAF.

## Introduction

Cystic fibrosis (CF) is the most common autosomal recessive genetic disease among white patients. It affects the exocrine glands, which, when producing abnormally viscous secretions, cause a series of manifestations, mainly respiratory and digestive.^1^, ^2^ CF is caused by a series of mutations in a gene that performs cystic fibrosis transmembrane conductance regulator (CFTR) encoding.^3^ Malfunction or absence of CFTR activity causes dehydration of the mucous secretions and an increase in its viscosity, favoring the obstruction of the ducts and consequent inflammation and fibrosis.^4^, ^5^, ^6^

Among the main agents for cochlear alteration through ototoxicity are AG (aminoglycoside) antibiotics, widely used in the treatment of CF to combat colonization by bacteria. Due to the frequent use of this type of drug, patients with cystic fibrosis are at high risk of developing hearing loss.^7^, ^8^, ^9^, ^10^

The hearing monitoring of patients exposed to ototoxic agents aims to detect hearing loss before the occurrence of impairment at the range of frequencies corresponding to speech, with consequent damage to communication; and to enable an early audiological intervention through individual hearing aids and speech therapy in those cases in which hearing loss is already evident.^11^, ^12^, ^13^ This monitoring is especially critical for the pediatric population, for even hearing loss limited to high frequencies can impair the child's language development.^14^ Currently, the most commonly used procedures to monitor hearing function in cases of ototoxicity are: pure tone audiometry (PTA), high frequency audiometry (HFA), and otoacoustic emissions (OAE).^13^

HFA evaluates pure tone air conduction thresholds in the 9–20 kHz range, depending on the equipment. This is an important test for the early detection of hearing loss caused by damage to the base of the cochlea, as occurs in cases of ototoxicity, because it allows the detection of hearing loss before reaching the conventionally measured frequency range (250–8000 Hz).^15^, ^16^

The literature presents some studies on CF patients evaluating hearing by HFA. Fausti et al.^17^ conducted PTA and HFA in 53 patients after treatment with AG. Hearing loss was detected in 47% of evaluated ears, and HFA alteration was primarily detected in 71% of ears. In previous studies, other authors found similar outcomes with high hearing thresholds in HFA following AG treatment in CF patients.^18^, ^19^ However, Mulheran et al.^20^ found that the risk for cochleotoxicity in CF is relatively low, approximately 2% for an intravenous antibiotic course. The authors suggested that the disease may attenuate the progression of cochleotoxicity, thanks to the rapid renal clearance of these drugs. Mulheran et al.^21^ reinforced this assumption with HFA, with absence of cochleotoxicity after treatment with tobramycin in CF patients. The authors highlighted the fact that the cumulative effect of AG was not assessed, because the aim of their study was to evaluate the cochleotoxicity of tobramycin administered one to three times per day. When assessing the prevalence of hearing loss and its relation to the use of antibiotics in CF, Cheng et al.^10^ observed hearing loss by PTA in 14% of 50 patients and identified the administration of ten or more courses of AG as a risk factor.

Examination by OAE does not require that the patient provides responses, and this test is very suitable for small children and even for adults unable to respond to tests such as audiometry.^22^ In addition, changes in OAE may reflect cochlear damage that is not as yet detectable by audiometry. Decreases in amplitude and in the dynamic area of responses and/or loss of distortion product OAE (DPOAE) response can result if there is a change in the function of outer hair cells.^23^

In a study of CF patients and a control group, Mulheran et al.^24^ observed normal hearing thresholds in both groups; however, these authors detected a significant increase of the stimulus required to generate DPOAE at 4 kHz in CF group. The authors suggested that this rise could represent one of the first alterations occurring in outer hair cells, caused by gentamicin. Stavroulaki et al.^8^ found that DPOAE is more sensitive than PTA in detecting cochlear changes after the use of gentamicin. Using DPOAE, other authors^25^, ^26^ also found cochlear alterations occurring prior to changes in hearing threshold. However, some authors, in their studies with ototoxic agents, claim that HFA is more effective than OAE to detect hearing loss.^23^, ^27^, ^28^, ^29^

In Brazil, there are few studies on CF patients’ hearing utilizing high frequency audiometry. Therefore, this study aimed to evaluate hearing thresholds testing high frequencies and using DPOAE in CF patients from this institution.

## Methods

This study was approved under No. 120096 by the Ethics Committee of the institution.

This was an observational, cross-sectional study. The population studied was composed of a study group (SG) with 39 children and adolescents referred from adult and pediatric cystic fibrosis (CF) outpatient clinics from this institution. The control group (CG) comprised 36 children and adolescents from the institution's otorhinolaryngology outpatient clinic, trainees, and children of employees of the institution and students of a state elementary school.

The inclusion criteria for the study group were as follows: individuals with a confirmed diagnosis of CF, aged 7–20 years, treated at the CF outpatient clinic, with type A tympanometric curve^30^ compatible with a middle ear without otological abnormalities. For the control group, inclusion criteria were individuals aged 7–20 years without otological and auditory complaints and with an A type tympanometric curve.^30^

Exclusion criteria for study and control groups were as follows: individuals with illnesses associated with ear repercussion (*e.g.*, meningitis, acoustic trauma, tinnitus, otorrhea, history of recurrent otitis media in early childhood), family history of hearing loss, type B or C tympanometric curve,^30^ and/or refusal to sign the informed consent (Appendix I). The history of diseases associated with ear repercussion was verified by reviewing medical records.

### Instruments and measures

The protocol followed to carry out the tests was the same used in the otorhinolaryngology and speech therapy outpatient clinic from the institution. The tests were performed by two trained speech therapists.

Initially, the subject was submitted to an ear evaluation by an otorhinolaryngologist. Then, tests of acoustic impedance, pure tone audiometry, high frequency audiometry, and product-distortion otoacoustic emissions were conducted. After the tests, the patient's medical record was analyzed in order to collect data relevant to the study.

The impedancemetry examinations were performed with the AZ26 Impedancemeter (Interacoustics – Denmark), with tympanometry and a survey of contralateral acoustic reflexes. The pure tone and high frequency audiometry tests were performed with the Siemens Unity PC audiometer (Germany), calibrated in dB HL according to ANSI standard 3.6-1989 with the use of HDA 2000 air and bone conduction B-71 phones. Pure tone audiometry by air and bone conduction was performed at 250–16,000 Hz and at 500–4000 Hz, respectively. All audiological tests were performed by two trained speech therapists using the same evaluation protocol.

The criteria of normality used for pure tone audiometry were the International Bureau for Audiophonology (Bureau International d’Audiophonologie [BIAP]) classification, in which the average of the frequencies of 500, 1000, 2000, and 4000 Hz <20 dB HL is considered as normal hearing. For high frequencies (9000–16,000 Hz), thresholds up to 25 dB HL were used as a criterion of normality, as documented in previous studies of normal hearing individuals.^31^, ^32^, ^33^, ^34^

The distortion-product otoacoustic emissions test was conducted in an acoustically treated room with the ILO 292 system (Otodynamics – England). The examination was performed in the frequencies of 1000, 1500, 2000, 3000, 4000, and 6000 Hz. A probe was inserted into the patient's ear, with an audible stimulus simultaneously generated by two pure tones of different frequencies (F1 and F2), in which F1 = 65 dB, F2 = 55 dB, and F2/F1 = 1.22. The test was analyzed by a DP-gram chart, and presence of response was considered as the finding of amplitudes equal to or greater than 3 dB SPL above the noise level.^35^

### Statistical analysis

Quantitative variables were expressed as mean and standard deviation or median and interquartile range. Categorical variables were described by absolute and relative frequencies.

To compare means between groups, Student's *t*-test for independent samples was applied. In the case of asymmetry, the Mann–Whitney test was used.

In the comparison of proportions, Fisher's exact test was used.

The significance level was set at 5% (*p* ≤ 0.05) and analyses were performed using SPSS software, version 18.0.

## Results

The sample consisted of 75 subjects: SG = 39 and CG = 36. The mean age was 13.0 (±3.2) and 12.3 (±4.1) years for the SG and CG, respectively. The participants’ age range was 7–20 years. Twenty-two subjects in the SG (56.4%) and 12 in the CG (33.3%) were male. Table 1 shows the results of the descriptive analysis of age and gender variables for each group, genetic mutations in CF, and number of intravenous AG cycles received by SG participants.

**Table 1:** Sample characterization.

Variable	Study group (*n* = 39)	Control group (*n* = 36)	*p*-Value
*Age (years), mean* *±* *SD*	13.0 ± 3.2	12.3 ± 4.1	0.420^a^

*Age group, n (%)*			0.223^b^
<12 years	11 (28.2)	16 (44.4)	
12–17 years	24 (61.5)	15 (41.7)	
>18 years	4 (10.3)	5 (13.9)	

*Gender, n (%)*			0.076^b^
Male	22 (56.4)	12 (33.3)	
Female	17 (43.6)	24 (66.7)	

*Mutation, n (%)*			–
Delta F508	23 (92.0)	–	
R553X	1 (4.0)	–	
L543X and A561E	1 (4.0)	–	

*AG courses, n (%)*			–
<10	33 (84.6)	–	
>10	6 (15.4)	–	

SD, standard deviation; AG, aminoglycosides.

a Student's *t*-test.

b Chi-squared test (Pearson).

There was no statistically significant difference in age and gender variables between the SG and CG, indicating that the subjects were similar in these aspects. Therefore, the sample was analyzed without reference to these variables. In the analysis of auditory thresholds between right and left ear, a statistically significant difference was noted only in the frequency of 250 Hz in the SG; in other frequencies and in the CG there was no such a difference. For this reason, the ears were analyzed with the use of their means at each frequency.

When auditory thresholds were compared between groups, the SG had significantly higher thresholds at 250 Hz, 1000 Hz, 8000 Hz, 9000 Hz, 10,000 Hz, 12,500 Hz, and 16,000 Hz. The mean values at 250–8000 Hz and at 9000–16,000 Hz were significantly higher in the SG (Table 2).

**Table 2:** Comparison of hearing thresholds (dB HL) between groups.

Frequency (Hz)	Study group (*n* = 39)	Control group (*n* = 36)	*p*-value^a^
	Md (P25–P75)	Md (P25–P75)	
250	5 (2.5–7.5)	5 (0–5)	0.036
500	5 (2.5–10)	5 (2.5–7.5)	0.055
1000	2.5 (0–5)	2.5 (0–2.5)	0.042
2000	5 (2.5–7.5)	3.8 (0–5)	0.092
3000	5 (2.5–7.5)	5 (2.5–5)	0.444
4000	5 (2.5–7.5)	5 (2.5–6.9)	0.313
6000	7.5 (5–12.5)	7.5 (5–10)	0.533
8000	7.5 (5–15)	5 (2.5–7.5)	0.003
Mean 250–8000 Hz	5.9 (3.4–8.1)	4.4 (2.3–5.9)	0.016
9000	5 (2.5–15)	5 (2.5–7.5)	0.022
10,000	5 (2.5–10)	2.5 (0–5)	0.002
11,200	7.5 (2.5–15)	5 (2.5–7.5)	0.096
12,500	5 (2.5–10)	2.5 (0–5)	0.034
14,000	2.5 (0–12.5)	0 (0–2.5)	0.160
16,000	2.5 (0–22.5)	0 (0–0)	<0.001
Mean 9000–16,000 Hz	4.6 (2.9–11.7)	2.9 (1.8–5.0)	0.005

a Mann–Whitney test.

**Figure 1:**
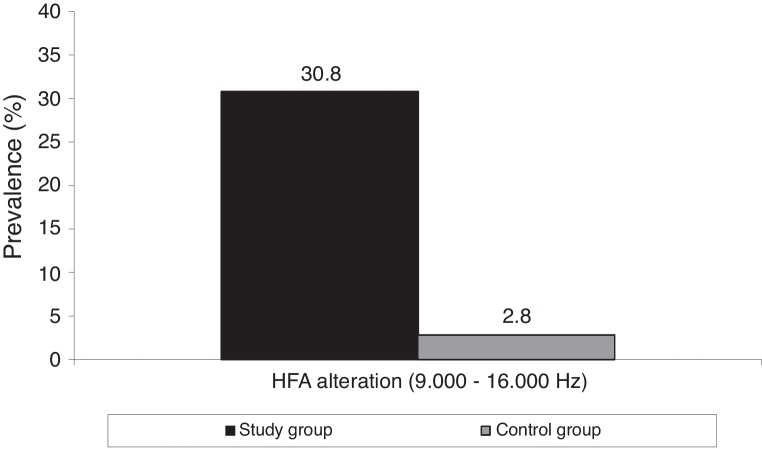
Alterations in HFA in study and control groups. HFA, high frequency audiometry.

The SG showed a higher prevalence of hearing loss by HFA (*p* = 0.004) (Fig. 1). Using PTA in the range of 250–8000 Hz, neither group exhibited abnormalities.

**Figure 2:**
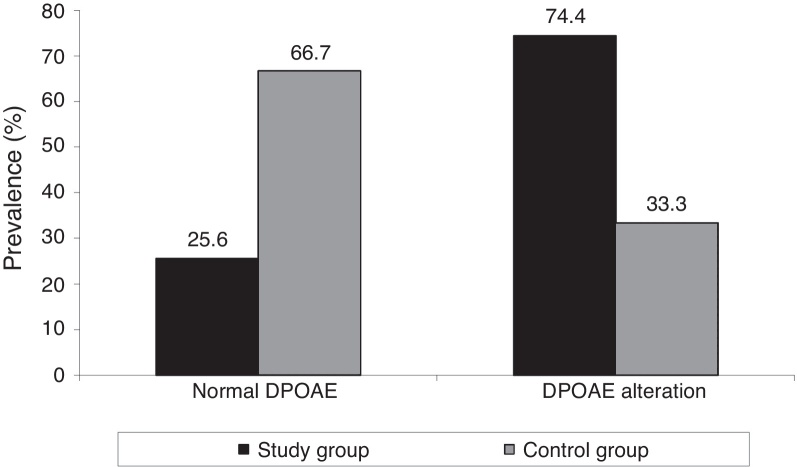
DPOAE in study and control groups. DPOAE, distortion-product otoacoustic emissions.

The SG showed a significantly higher prevalence of DPOAE alteration when compared to the CG (*p* = 0.001), as shown in Fig. 2.

The SG presented more DPOAE alterations at 1000 Hz and 6000 Hz in both ears (Table 3), when comparing right and left ears at each frequency and for each group. No significant difference between the ears was noted (*p* > 0.20).

**Table 3:** Comparison of alterations in DPOAE by frequency and by ear between groups.

Frequency (Hz)	Ear	Study group (*n* = 39)	Control group (*n* = 36)	*p*-Value^a^
1000	Right	10 (25.6)	2 (5.6)	0.040
	Left	8 (20.5)	1 (2.8)	0.029
1400	Right	3 (7.7)	1 (2.8)	0.616
	Left	3 (7.7)	0 (0.0)	0.241
2000	Right	5 (12.8)	0 (0.0)	0.055
	Left	2 (5.1)	2 (5.6)	1.000
2800	Right	6 (15.4)	3 (8.3)	0.483
	Left	5 (12.8)	1 (2.8)	0.202
4000	Right	6 (15.4)	2 (5.6)	0.265
	Left	9 (23.1)	3 (8.3)	0.154
6000	Right	13 (33.3)	1 (2.8)	0.002
	Left	16 (41.0)	3 (8.3)	0.003

a Fisher's exact test.

When comparing DPOAE amplitudes between groups, the SG had lower amplitudes at 1000 Hz, 1400 Hz, and 6000 Hz (Table 4). When comparing left and right ears with respect to DPOAE amplitude, there was significant difference only at 4000 Hz in the SG (*p* = 0.037).

**Table 4:** Comparison of amplitudes (dB SPL) of DPOAE between groups.

Frequency (Hz)	Ear	Study group (*n* = 39)	Control group (*n* = 36)	*p*-Value^a^
		Md (P25–P75)	Md (P25–P75)	
1000	Right	10.1 (5.5–15.3)	11.2 (8.9–17.6)	0.168
	Left	9.2 (5.5–15.2)	13.3 (9.6–7.2)	0.028
1400	Right	16.0 (6.8–19.0)	15.6 (13.2–19.1)	0.528
	Left	14.5 (6.9–18.2)	16.2 (13.0–20.2)	0.041
2000	Right	12.6 (6.5–16.9)	14.2 (8.5–18.8)	0.233
	Left	10.8 (6.4–16.0)	14.1 (7.8–18.8)	0.051
2800	Right	11.2 (6.7–15.3)	12.4 (5.4–16.1)	0.451
	Left	10.2 (4.5–13.8)	11.0 (7.4–15.7)	0.148
4000	Right	9.2 (2.0–14.8)	14.4 (4.7–17.6)	0.166
	Left	10.2 (1.4–13.8)	11.2 (7.4–16.6)	0.072
6000	Right	5.6 (−3.2 to 12.2)	8.7 (2.0–12.7)	0.102
	Left	3.6 (−4.1 to 10.2)	9.7 (4.4–14.3)	0.007

dB SPL, decibel sound pressure level.

a Mann–Whitney test.

**Figure 3:**
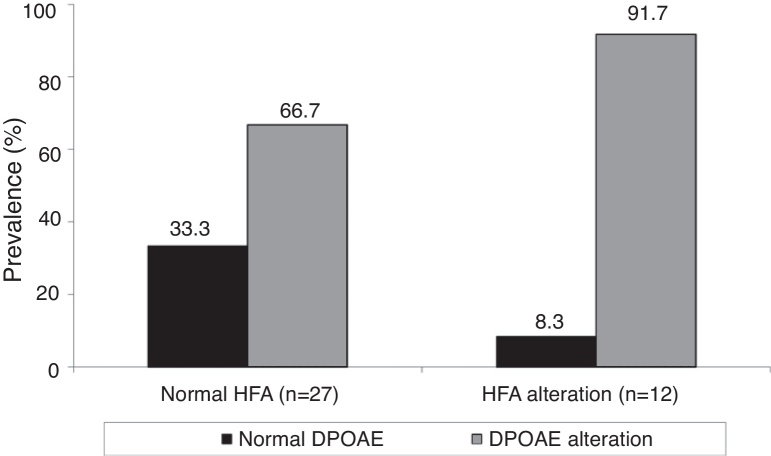
Association between HFA and DPOAE alterations in the study group. HFA, high frequency audiometry; DPOAE, distortion-product otoacoustic emissions.

We found that DPOAE alterations occurred when testing high frequencies, both in the study group with normal HFA (66.7%) and in the group with HFA alterations (91.7%), albeit more often in the latter. However, the statistical analysis showed that there was no association between changes in HFA and in DPOAE for SG participants (*p* = 0.131) (Fig. 3).

**Figure 4:**
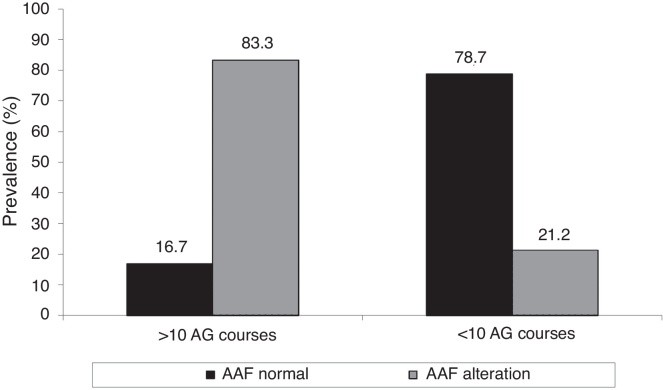
Association between number of AG cycles and HFA alteration. AG, aminoglycosides; HFA, high frequency audiometry.

There was a significant association between changes in hearing thresholds by HFA with number of AG courses administered (*p* = 0.005). The administration of more than ten courses of intravenous AG was associated with a higher prevalence of HFA alterations (Fig. 4).

## Discussion

HFA is described as the most effective method for early detection of cochlear alterations due to ototoxicity.^23^, ^29^, ^36^ Its use is still limited, due to lack of standardization of calibration and of normality parameters,^16^ particularly in the pediatric population, because of low test reliability in children under 7 years of age.^37^

Conversely, evoked otoacoustic emissions is a quick and objective test, showing a great advantage for both pediatric and adult population, who often fail to respond appropriately to audiometry tests, due to the disease and its treatment.^22^, ^38^ Considering the fact that otoacoustic emissions may exhibit alterations even in individuals with normal thresholds in the face of an early impairment of outer hair cells, this procedure is widely used in ototoxicity monitoring.^11^, ^39^

Considering that one of the major causes of ototoxicity is the use of aminoglycosides, and that CF patients receive repeated courses of this type of drug, this population is at high risk of developing cochlear alterations.^7^, ^8^, ^9^, ^10^

By analyzing the sample, there was no significant difference in male *versus* female thresholds, as Abujamra et al. also found.^29^ Although these authors have studied the effects of other type of ototoxic drug (cisplatin), they also verified no significant differences.

Neither was a significant difference detected in right *vs.* left ear thresholds, and for this reason the ears’ mean was used to represent such thresholds. In studies with normal hearing individuals, Sahyeb et al.^32^ and Sá et al.^33^ also detected no difference between the ears using PTA and HFA.

When comparing hearing thresholds obtained by PTA (250–8000 Hz) and HFA (9000–16,000 Hz) between groups, significantly higher thresholds were observed in the study group, with the largest differences occurring in HFA. Hearing loss was not observed in PTA; however, HFA detected high prevalence of hearing loss in the study group. These findings demonstrate the importance of using HFA in monitoring CF patients.^19^, ^40^

These results differ from those obtained by Martins et al.,^41^ who observed hearing loss at conventional frequencies and at high frequencies in their patients. However, Al-Malky et al.^42^ found hearing loss only at 8000–20,000 Hz by HFA and reduced amplitudes using DPOAE, which agrees with the present findings. In that study, the authors highlight the fact that although the hearing loss is associated with high AG exposure, it only occurred in 21% of the exposed group. Faced with this fact, the authors suggest that there may be other factors responsible for hearing loss.

In the present study, higher number of alterations by frequency and also lower amplitudes in DPOAE were noted in the SG *vs.* CG, even with normal hearing thresholds up to 8000 Hz. This finding supports the study of Stavroulaki et al.,^8^ who found decreased amplitudes of DPOAE at frequencies above 3000 Hz in children with CF after treatment with gentamicin. Mulheran et al.,^24^ Katbamna et al.,^43^ and Katbamna et al.^44^ obtained similar outcomes, but these authors used different assessment methods of DPOAE.

When trying to establish a potential association between alterations in DPOAE and alterations in HFA, since the alterations in DPOAE occurred more frequently in individuals with alterations in HFA, this association was not found. This result may be related to the size of the sample. This finding disagreed with that of Arnold et al.,^45^ who concluded that hearing at high frequencies significantly influences DPOAE at lower frequencies. However, that study was conducted with healthy, normal-hearing young adults.

With respect to AG effects, it is known that repeated courses of treatment and its duration are factors related to increased risk of ototoxicity.^46^, ^47^ In the present study we observed a significant association between receiving more than ten intravenous AG courses and HFA alterations. Some authors found similar results,^10^, ^19^, ^24^ while in another study the findings were different.^40^ In their study, Mulheran et al.^20^ concluded that there is possibly an increased risk of ototoxicity related to the number of AG courses taken, but the authors point out that this relationship is not linear. Due to the low prevalence of ototoxicity found in CF patients *vs.* individuals without the disease who were treated with AG, some authors^20^, ^48^ suggest that CF itself is a protective factor for ototoxicity. These authors suggest that such protection may be due to altered pharmacokinetics and/or underlies changes in CFTR, but that more research is needed in this area. Furthermore, it is known that CF results in a faster renal clearance of drugs, including AG,^49^, ^50^ and this may also contribute to reduced ototoxicity.

A significantly higher prevalence of DPOAE alterations in SG, with the presence of more alterations at 1000 and 6000 Hz in both ears and with lower amplitudes at 1000, 1400, and 6000 Hz, suggest that this test should also be conducted in CF patients, even in those with normal hearing. The significantly elevated hearing thresholds at 250; 1000, 8000, 9000, 10,000, 12,500, and 16,000 Hz and the exclusive presence of HFA alterations in 30.8% of the SG suggest that this assessment should be incorporated into the auditory monitoring of CF patients.

## Conclusion

This study showed that a significant number of CF patients who received repeated AG courses demonstrated HFA and DPOAE alterations, and that there was a significant association between administration of more than ten intravenous AG courses and a higher prevalence of HFA alterations.

## Funding

This study was funded by 10.13039/501100003593CNPq.

## Conflicts of interest

The authors declare no conflicts of interest.

## Figures and Tables

**Table:** 

**Free and informed consent form – Study Group**
We are inviting you and/or your child to participate in the study “High frequency hearing thresholds and product-distortion otoacoustic emissions in cystic fibrosis patients.” This study aims to study hearing tests that can show if hearing loss occurs in patients with cystic fibrosis, even if their hearing difficulty is not perceived.
You and/or your child are being invited to participate in the research study group, that is, the group of participants who have cystic fibrosis.
Routine examinations will be conducted: pure tone audiometry, impedancemetry, and otoacoustic emissions. The total duration of the three tests is approximately 40 minutes.
Compared to routine audiometry, the difference in the audiometry that will be carried out is that a wider range of sounds will be evaluated. We will evaluate higher-pitch sounds (very high tones), making it a more comprehensive examination. The main benefit of this procedure is to establish an early diagnosis of hearing loss before the frequencies used in the communication, i.e., the frequencies of speech, are affected. To perform this test, the patient is placed inside a soundproof booth with headphones in their ears; the patient will hear whistles and should tell the audiologist when they are hearing the sound. The examination has an average duration of 30 minutes.
In the impedancemetry test, a headphone is placed in one of the patient's ears, and in the other ear a probe is placed, causing slight pressure and sending some whistles. The average duration of this test is five minutes.
In the otoacoustic emissions test, a small probe is placed in the ear; the probe sends some sounds. The average duration of this test is five minutes.
These three tests are painless and well tolerated by most patients, but if there are any unpleasant sensations, any of them may be discontinued at once.
The participant and/or guardian has the right to request information about the study before it is performed and during its realization. The participant and/or guardian also has the right to refuse to participate or withdraw their consent at any stage of the study, without any penalty and with no prejudice to the participant's care.
The participant's privacy will always be preserved, as well as his/her sensitive data. If the study is published, the participant's name will not be revealed.
There will be no cost to the participant and/or guardian of his/her participation.
A copy of this document will be delivered to the participant and/or guardian.
Head researcher: Physician, Pulmonology Department of institution. Contact phone number: (51) 3359-8943
Participating researchers: Speech therapist at the Otorhinolaryngology Department of the institution, zone 19. Contact phone number: (51) 3359 82 28
Participating researchers: Speech therapist at the Otorhinolaryngology Department of the institution, zone 19. Contact phone number: (51) 3359 82 28
Ethics and Research Committee of the institution (for questions about ethical issues only) Phone: (51) 3359 83 04
Date: ___ / ____ / ____ Participant name: _________________________________________ Name of parent/guardian: ____________________________________ Parent/guardian signature: _________________________________ Researcher name: ______________________________________ Researcher signature: ___________________________________
